# On the Use of Calomel as a Vermifuge

**Published:** 1807-05

**Authors:** 


### *To the Editors of the Medical and Physical Journal.*

GENTLEMEN,

WITH all possible deference for the decision of the learned Coroner of Hull, and his Jury, aided by the professional knowledge of Messrs. John and James Sagner, I will solicit your permission to enter my protest against the conclusion drawn by Mr. Thomas Clayton, in the case of his child, communicated by *Aminicus*, and inserted in your 96th Number, page 173.

Can it be possible for two or three grains of calomel, the utmost quantity, I should presume, contained in the dose for a child of that age, if the printed directions were properly properly attended to; can it be possible, I would ask, for that dose of calomel, to produce in a fine boy, three years old, the symptoms related by Mr. Clayton? My own experience in nearly forty years practice and a liberal use of calomel to children, does not warrant such a conclusion.

That the poor child has fallen a victim to the injudicious administration of an active remedy, at an improper point of time, is highly probable; but the case is far too vaguely related, to enable any man to form a clear opinion of the state of the child, at the period when the father thought proper to give the mercurial lozenge. This, it must be admitted, is a most important circumstance, such as would require minute investigation, and serious deliberation, before a conscientious practitioner of mature judgment, could subscribe so positive a declaration as that contained in the verdict, "POISONED BY CHING'S WORM LOZENGES." It may, however, be reasonably imagined, that some previous indisposition in the child, induced the father to have recourse to the quack medicine; and, from the relation he has given of the symptoms, I am strongly inclined to believe the case required bark, steel, and the diluted acids, instead of repeated purging.

It will be readily granted, that mercury is a poison equally capable with arsenic of destroying life; and so is tartarised antimony, opium, digitalis, and a variety of other remedies, which skilful and experienced practitioners are daily prescribing with the most salutary effect. These, then, strictly speaking, are only poisons in degree, and are never dangerous but in the hands of ignorance or despair.

Most devoutly is it to be wished, that the fate of this unfortunate child may operate as a caution to parents, and those who have the care of children, and check the prevalent and increasing use of quack medicines, as well as restrain the frequent and fatal exhibition of antimonials, mercurials, and opiates, which are all medicines of great power, and yet are daily prescribed at random, by lady doctors, and quacking persons. The power and efficacy of a remedy do but increase the danger attending its unskilful administration. Pity it is that people are not impressed with a firm conviction of this truth, that extensive knowledge, great experience and observation, sound judgment, and critical inquiry, are indispensible requisites to the successful practice of medicine.

A.

*London, Feb.* 12, 1807.

